# Arsenic Exposure and Motor Function among Children in Bangladesh

**DOI:** 10.1289/ehp.1103548

**Published:** 2011-07-08

**Authors:** Faruque Parvez, Gail A. Wasserman, Pam Factor-Litvak, Xinhua Liu, Vesna Slavkovich, Abu B. Siddique, Rebeka Sultana, Ruksana Sultana, Tariqul Islam, Diane Levy, Jacob L. Mey, Alexander van Geen, Khalid Khan, Jennie Kline, Habibul Ahsan, Joseph H. Graziano

**Affiliations:** 1 Department of Environmental Health Sciences, Columbia University Mailman School of Public Health, New York, New York, USA; 2 Department of Psychiatry, College of Physicians and Surgeons, Columbia University, New York, New York, USA; 3 New York State Psychiatric Institute, New York, New York, USA; 4 Department of Epidemiology, and; 5 Department of Biostatistics, Columbia University Mailman School of Public Health, New York, New York, USA; 6 University of Chicago and Columbia University Arsenic Project Office, Mohakhali, Dhaka, Bangladesh; 7 Lamont-Doherty Earth Observatory of Columbia University, Palisades, New York, USA; 8 Department of Health Studies, University of Chicago, Chicago, Illinois, USA

**Keywords:** arsenic, Bangladesh, bodily coordination, fine motor control, manganese, motor function, neurotoxicity, selenium

## Abstract

Background: Several reports indicate that drinking water arsenic (WAs) and manganese (WMn) are associated with children’s intellectual function. Very little is known, however, about possible associations with other neurologic outcomes such as motor function.

Methods: We investigated the associations of WAs and WMn with motor function in 304 children in Bangladesh, 8–11 years of age. We measured As and Mn concentrations in drinking water, blood, urine, and toenails. We assessed motor function with the Bruininks-Oseretsky test, version 2, in four subscales—fine manual control (FMC), manual coordination (MC), body coordination (BC), and strength and agility—which can be summarized with a total motor composite score (TMC).

Results: Log-transformed blood As was associated with decreases in TMC [β = –3.63; 95% confidence interval (CI): –6.72, –0.54; *p* < 0.01], FMC (β = –1.68; 95% CI: –3.19, –0.18; *p* < 0.05), and BC (β = –1.61; 95% CI: –2.72, –0.51; *p* < 0.01), with adjustment for sex, school attendance, head circumference, mother’s intelligence, plasma ferritin, and blood Mn, lead, and selenium. Other measures of As exposure (WAs, urinary As, and toenail As) also were inversely associated with motor function scores, particularly TMC and BC. Square-transformed blood selenium was positively associated with TMC (β = 3.54; 95% CI: 1.10, 6.0; *p* < 0.01), FMC (β = 1.55; 95% CI: 0.40, 2.70; *p* < 0.005), and MC (β = 1.57; 95% CI: 0.60, 2.75; *p* < 0.005) in the unadjusted models. Mn exposure was not significantly associated with motor function.

Conclusion: Our research demonstrates an adverse association of As exposure and a protective association of Se on motor function in children.

Epidemiologic evidence of neurotoxicity associated with arsenic (As) exposure from occupational sources or via drinking water is well documented (Hafeman et al. 2005; Mukherjee et al. 2003; Rahman et al. 2001; Tseng et al. 2006). In the past decade, studies from China (Wang et al. 2007), Mexico (Calderon et al. 2001; Rosado et al. 2007), India (von Ehrenstein et al. 2007), and Taiwan (Tseng et al. 2006) have also demonstrated evidence of an impact on cognitive development, reflected in lower intelligence scores, deficits in long-term memory, and delayed linguistic abstraction among children exposed to As from drinking water. We previously described lower intellectual functioning among children exposed to low to moderate levels of drinking water As (WAs) in two separate studies in Bangladesh (Wasserman et al. 2004, 2007). Recent studies have also reported evidence of manganese (Mn)–induced neurotoxicity in children (Bouchard et al. 2007; Wasserman et al. 2006; Wright et al. 2006). For instance, we observed deficits in intellectual functioning among children exposed to elevated Mn in drinking water in Bangladesh (Wasserman et al. 2006), and in Korea children with higher blood Mn (BMn) received lower intelligence test scores (Kim et al. 2009). In a small study of U.S. children living on a former mining site, those with higher hair As and Mn performed less well in cognitive tests (Wright et al. 2006).

Until now, most epidemiologic studies have focused on impaired cognitive function as a measure of neurotoxicity in children exposed to As. Other developmental outcomes, such as motor function, have largely been ignored. Several reports have noted evidence of As-induced neurotoxicity, including peripheral neuropathy, in adults (Hafeman et al. 2005; Mukherjee et al. 2003). A recent cross-sectional study of Taiwanese adolescents reported adverse associations between WAs and several measures of motor and sensory nerve peripheral conduction (Tseng et al. 2006).

In 2000, we established the Health Effects of Arsenic Longitudinal Study (HEALS) of 12,000 adults in Araihazar, Bangladesh, and have studied subsets of their children who have naturally occurring As and Mn in their drinking water; in 2006–2008 an additional 8,000 participants were added to the cohort. In the present study, we examined whether As and Mn from drinking water were associated with motor function in 8- to 11-year-old children. Because selenium (Se) is known to antagonize As (Levander 1977), we also measured blood Se (BSe) and examined its role in motor function among our study participants.

## Materials and Methods

*Participants.* We obtained approval from institutional review boards at Columbia University and from the Bangladesh Medical Research Council. A detailed description of the methodology, including selection and enrollment procedures, water, and biological sample collection and processing, has been previously published (Wasserman et al. 2011). In short, our aim was to recruit 75 eligible children 8–11 years of age into each of four groups based on their home well-water concentrations: high As–high Mn (As > 10 μg/L and Mn > 500 μg/L), high As–low Mn, low As–high Mn, and low As–low Mn.

*Procedure.* We used data on WAs and drinking water Mn (WMn) from our HEALS cohort central database to generate a list of 772 households with children who were potential participants. Between January and December 2008, field staff verified their current address, As and Mn status of their well water, and eligibility criteria. If parents and children were willing, and if the child was attending school in an age-appropriate class, informed parental consent and child assent were obtained. At this visit, the field team also collected sociodemographic information for the household and made an appointment for mother and child to appear at the field clinic. We continued household visits until approximately 75 children in each of the four groups had been recruited. At the field clinic, motor function was assessed using the Bruininks-Oseretsky test, 2nd edition (BOT-2) (Bruininks and Bruininks 2005). Biological samples (urine, blood, and toenail samples) were collected.

In total, 304 children completed assessments. Five participants were excluded from analysis because of inadequate blood samples.

*Measures.* Water sample collection and measurement of As and Mn. Children who had been drinking water from the same well for at least the past year were recruited. At the time of recruitment, almost all (*n* = 297, 97.7%) mothers of children in the current cohort had reported drinking exclusively from the index well, and 78% had drunk water from that well for ≥ 5 years.

Field sample collection and laboratory analyses procedures are described elsewhere in detail (Cheng et al. 2004; van Geen et al. 2005, 2007). Water samples were analyzed by high-resolution inductively coupled plasma mass spectrometry (HR-ICP-MS) as previously described (van Geen et al. 2007). The analytical detection limit of the method is 0.1 μg/L; the standard deviation of a single measurement is conservatively estimated at 4 μg/L (van Geen et al. 2005). Mn concentrations were also determined by HR-ICP-MS. The detection limit of the method for Mn is also 0.1 μg/L, and its precision is 2% (Cheng et al. 2004).

Water samples were collected in 20-mL polyethylene scintillation vials. The samples were acidified to 1% with high-purity Optima HCl (Fisher Scientific, Pittsburg, PA, USA) at least 48 hr before analysis (van Geen et al. 2007). Water samples are then diluted 1:10 for As and Mn by HR-ICP-MS (Cheng et al. 2004; van Geen et al. 2005). For As, the detection limit of the method is typically < 0.2 μg/L, and the long-term reproducibility determined from consistency standards included with each run averages 4% (1 – σ) in the 40–500 μg/L range. For Mn, the detection limit of the method is typically < 0.02 mg/L, and the long-term reproducibility averages 6% in the 0.2–2.0 mg/L range.

Urine collection and urinary As and creatinine assays. Spot urine samples were collected in 50-mL acid-washed tubes and carried in portable coolers with ice packs for up to 6 hr until storage at –20°C. All samples were frozen until shipment to Columbia University on dry ice. Urinary As (UAs) assays were performed with graphite furnace atomic absorption using a PerkinElmer Analyst 600 graphite furnace system (PerkinElmer, Shelton, CT, USA) as previously described (Nixon et al. 1991). The detection limit for UAs was 2 μg/L. Urinary creatinine (UCr) was analyzed by a colorimetric method based on the Jaffe reaction.

Blood sample collection and processing. Blood samples were collected in two Vacutainers with EDTA to prevent coagulation. One vacutainer was frozen intact, whereas plasma from the other was aliquoted; all were kept at –20°C until shipment to Columbia University on dry ice.

Venous whole blood samples were analyzed for blood lead (BPb), BMn, BSe, and blood As (BAs) using a PerkinElmer Elan DRC (Dynamic Reaction Cell) II ICP-MS equipped with an AS 93+ autosampler (PerkinElmer). ICP-MS-DRC methods for metals in whole blood were developed according to published procedures (Pruszkowski et al. 1998; Stroh 1988), with modifications for blood sample preparation as suggested by the Laboratory for ICP-MS Comparison Program, Institut National de Sante Publique du Quebec. Hemoglobin and plasma ferritin (Miles et al. 1974) were also measured.

Toenail collection and assay. Nail collection, washing, and digestion were performed using a combination of two published methods (Chen et al. 1999; Das et al. 1995). After collection, the toenail samples were thoroughly washed, dried overnight, weighed, and digested in concentrated HNO_3_. The digested nail samples, diluted to final acid volume of 10%, were analyzed for As and Mn using a PerkinElmer Elan DRC II ICP-MS equipped with an AS 93+ autosampler (PerkinElmer). An ICP-MS-DRC method for metals in nails was developed from a published method (Pruszkowski et al. 1998), with modifications and adjustments based on suggestions from the PerkinElmer application laboratory. During the period in which all samples of this study were analyzed, the intraprecision coefficient of variations for nail As (NAs) and nail Mn (NMn) in these quality control samples were 2.4 and 1.0, respectively. Interprecision coefficients of variation for the same quality control samples for As and Mn were 5.3 and 6.1, respectively.

*Maternal intelligence.* Maternal intelligence was measured on the Wechsler Abbreviated Scale of Intelligence (WASI; 1999). Pilot testing indicated that many mothers without education were unable to master the abstract nature of the Similarities and Block Design subscales; thus, only the Vocabulary and Matrix Reasoning subscales were used (for more information, see Wasserman et al. 2011).

Motor function. The Bruininks-Oseretsky test, 2nd edition (BOT-2), is an individually administered test that measures a wide range of motor skills in young persons, standardized on a U.S. nationally representative sample of > 1,500 individuals 4–21 years of age. It is the most widely used standardized measure of motor proficiency, with excellent psychometrics reliability (0.90–0.97) and validity (positive predictive value ~ 88%) (Cairney et al. 2009; Deitz et al. 2007; Rodger et al. 2003; Wang TN et al. 2009; Wilson et al. 1995). It has been used successfully in studies of individuals with developmental coordination disorder (Rodger et al. 2003; Wilson et al. 1995); scores for children with developmental coordination disorder are between one and two standard deviations below norms (Deitz et al. 2007). The BOT-2 uses a composite structure organized around both the muscle groups and the limbs involved in movement, generating four subscale scores and a summary, Total Motor Composite (TMC). The Fine Manual Control (FMC) subscale examines coordination of the hands and fingers; the Manual Coordination (MC) subscale encompasses coordination of arms and hands, especially for object manipulation; the Body Coordination (BC) subscale considers posture and balance; and the Strength and Agility (SA) subscale considers locomotion.

Test materials were translated into Bangla and then back-translated into English. Study testers were trained by an experienced psychometrician (G.A.W.). Fifty children were tested in initial feasibility studies and were not included in this analysis. Testers were blind to exposure characteristics of study participants.

Questionnaire data on covariates. During home visits, the field team collected sociodemographic information, including living conditions, maternal and paternal education and occupation, school attendance, and other potential covariates (e.g., birth order, sibship size). A general assessment of child health was assessed during the home visit, via maternal interview.

*Clinical examinations, anthropometric data, and tests of mother’s intelligence.* A full clinical examination and history of any long-term illness was collected by a trained physician at the time of the field clinic visit. Height, weight, and head circumference were measured before the assessment of motor function. At the same visit, mother’s intelligence was estimated on the WASI.

*Statistical analysis.* Summary statistics were calculated to describe the sample characteristics. Chi-square tests and analysis of variance (ANOVA) were used to detect group differences in categorical and continuous variables. WAs, WMn, BAs, BMn, BPb, UAs, and UCr, were log transformed to normalize their distributions to meet assumptions of ANOVA and reduce the impact of extreme values in the linear regression analysis, whereas BSe was square transformed. Spearman correlation coefficients were used to evaluate bivariate associations among motor function subtests (FMC, MC, BC, SA), total score (TMC), and the exposure variables. We estimated associations between BAs and BMn and scores for each motor function subtest in separate linear regression models, with and without adjusting for sociodemographic characteristics and other potential confounders [sex, months of school attendance, head circumference, maternal WASI score, and plasma ferritin, based on an association with any of the four subtests or total score (*p* < 0.10)]. We also checked whether associations with BAs and BMn were altered by adjustment for BPb and BSe. When estimating associations with UAs, we used models with and without adjustment for UCr. We also examined interactions between BAs and BMn and for each measure of motor function by including multiplicative interaction terms in the models. A *p*-value of < 0.05 was considered statistically significant. All statistical analyses were conducted using SAS software (version 9.2; SAS Institute Inc., Cary, NC, USA).

## Results

*Characteristics of the study participants.* We found no significant differences between the participating and nonparticipating families in the sociodemographic factors sex of the child, mother’s age and educational attainment, number of living siblings, land or television ownership, duration of well use, or WAs concentration (data not shown).

We found no significant differences across the four WAs/WMn groups with regard to demographic, anthropometric, or social characteristics (Table 1). The average WAs concentration of the study participants was 43 μg/L. The average plasma ferritin and hemoglobin levels of participants were 33 ng/mL and 12.5 g/dL, respectively. Children exposed to higher WAs (> 10 μg/L) had significantly higher UAs (49.4 vs. 106.6 μg/L; *p* < 0.0001), UCr (161.2 vs. 331.3 g creatinine; *p* < 0.0001), BAs (3.3. vs. 6.3 μg/L; *p* < 0.0001), and NAs (3.0 vs. 8.8 μg/g; *p* < 0.0001) than did children with WAs ≤ 10 μg/L. In contrast, children exposed to higher WMn (> 500 μg/L) did not have higher levels of BMn (14.5 vs. 15.0 μg/L; *p* = 0.17) but differed in NMn (27.0 vs. 33.8 μg/g; *p <* 0.05), indicating that BMn may not be a good biomarker of WMn exposure in this population. Scores on TMC and four subscales (FMC, MC, BC, and SA) did not vary significantly among the four exposure groups based on combined WAs and WMn exposures (Table 2). As expected, our study children performed lower than their U.S. agemates on all subscales.

**Table 1:** Study participant characteristics and exposure measures by WAs and WMn distribution.

WAs and WMn levels								
Characteristics and exposure measures	Overall (*n* = 303)	Low As–low Mn (*n* = 77)	Low As–high Mn (*n* = 74)	High As–low Mn (*n* = 73)	High As–high Mn (*n* = 79)	*p*-Value
Participants’ characteristics												
Male [*n* (%)]		50.0 (151)		61.0 (47)		48.6 (36)		43.8 (32)		45.5 (36)		0.13
Child age (years)		9.6 ± 0.7		9.5 ± 0.8		9.6 ± 0.73		9.8 ± 0.7		9.5 ± 0.8		0.14
Month attending school		42.0 ± 16.2		43.3 ± 18.52		42.4 ± 14.8		42.5 ± 14.0		39.8 ± 17.1		0.69
Grade		2.9 ± 1.2		3.0 ± 1.3		2.8 ± 1.2		3.0 ± 1.1		2.9 ± 1.2		0.37
Head circumference (cm)		49.3 ± 1.4		49.3 ± 1.2		49.3 ± 1.4		49.0 ± 1.3		49.7 ± 1.7		0.17
Mother’s WASI raw score		31.0 ± 10.6		32.0 ± 9.7		30.2 ± 10.6		31.5 ± 10.8		30.2 ± 11.2		0.41
Exposure measures												
Blood (μg/L)												
BAs		4.8 ± 3.2		3.2 ± 1.7		3.4 ± 1.7		6.2 ± 4.2		6.3 ± 3.0		< 0.0001
BMn		14.7 ± 3.7		14.5 ± 3.8		15.4 ± 4.1		14.3 ± 3.5		14.6 ± 3.2		0.49
BSe		104.9 ± 17.2		106.4 ± 16.2		103.6 ± 15.5		106.7 ± 18.0		103.0 ± 18.7		0.43
BPb		114.5 ± 37.2		123.9 ± 38.4		113.9 ± 42.4		100.9 ± 30.5		118.5 ± 33.1		0.0009
Water (μg/L)												
WAs		43.3 ± 73.6		2.3 ± 2.3		3.3 ± 2.7		97.3 ± 108.8		70.7 ± 58.1		< 0.0001
WMn		725.5 ± 730.5		202.1 ± 145.4		1111.1 ± 686.1		184.0 ± 146.1		1367.1 ± 692.6		< 0.0001
Urine												
UAs (μg/L)		78.0 ± 72.1		46.7 ± 35.8		52.3 ± 38.7		106.0 ± 93.0		107.0 ± 79.6		< 0.0001
UCr (mg/dL)		35.0 ± 24.1		35.0 ± 25.0		33.7 ± 24.4		36.8 ± 24.9		34.4 ± 22.6		0.97
UAs (μg/g creatinine)		246.5 ± 183.9		149.5 ± 74.4		173.3 ± 111.7		330.5 ± 247.4		332.0 ± 170.4		< 0.0001
NAs (μg/g)		5.9 ± 6.3		2.9 ± 2.5		3.0 ± 2.2		6.9 ± 7.0		10.6 ± 7.7		< 0.0001
Hemoglobin (g/dL)		12.4 ± 0.9		12.7 ± 1.1		12.2 ± 0.8		12.3 ± 1.0		12.4 ± 0.8		0.002
Plasma ferritin (ng/mL)		33.4 ± 17.8		31.5 ± 16.8		32.1 ± 17.5		36.9 ± 18.1		33.1 ± 18.8		0.19
Data are mean ± SD, except as noted.												

**Table 2:** BOT-2 scores (mean ± SD) by WAs and WMn category.

Motor function measure		WAs and WMn levels								
		Overall (*n* = 303)	Low As–low Mn (*n* = 77)	Low As–high Mn (*n* = 74)	High As–low Mn (*n* = 73)	High As–high Mn (*n* = 79)	*p*-Value
FMC		42.3 ± 8.6		43.7 ± 7.6		42.8 ± 9.6		41.7 ± 8.8		41.2 ± 8.2		0.26
MC		38.9 ± 7.3		39.7 ± 6.9		39.2 ± 7.8		38.4 ± 8.2		38.2 ± 6.4		0.56
BC		41.3 ± 6.0		42.7 ± 5.9		41.2 ± 6.0		41.2 ± 6.5		40.0 ± 5.7		0.07
SA		37.4 ± 3.7		37.7 ± 3.7		37.5 ± 3.5		37.3 ± 3.9		37.2 ± 3.8		0.81
TMC		160.0 ± 18.5		162.5 ± 17.3		161.2 ± 19.8		159.2 ± 19.8		157.2 ± 17.2		0.33

*Relationships among measures of exposure and outcomes.* The various measures of As exposure (WAs, BAs, UAs, NAs) were moderately correlated with each other (*r*-values between 0.52 and 0.67, *p* < 0.0001; data not shown). The measures of Mn exposure were not well correlated with each other (*r*-values between 0.05 and 0.15; data not shown). BAs was correlated positively with BMn (*r* = 0.12, *p* = 0.02) and negatively with BSe (*r* = –0.13, *p* < 0.01). BMn was negatively associated with BSe (*r* = –0.33, *p* < 0.0001).

As expected, the motor subscales were correlated with each other (*r*-values between 0.27 and 0.36, all *p* < 0.001) and with total score (*r*-values between 0.56 and 0.77, all *p* < 0.001; data not shown).

*Associations between markers of As exposure and motor function.* Male sex, head circumference, duration of school attendance, and plasma ferritin levels were positively associated with TMC and, with the exception of serum ferritin, were also positively associated with one or more subtests. Maternal intelligence, measured by the WASI, was not associated with TMC or the four subtests (data not shown).

In unadjusted models, we found significant inverse associations between BAs and TMC [β = –3.96; 95% confidence interval (CI): –7.38, –0.55; *p* < 0.01], FMC (β = –1.94; 95% CI: –3.55, –0.35; *p* < 0.01), and BC (β = –1.38; 95% CI: –2.50, –0.26; *p* < 0.01) (Table 3). Adjusting for covariates, BAs was inversely associated with TMC (β = –3.63; 95% CI: –6.72, –0.54; *p* < 0.01), FMC (β = –1.68; 95% CI: 3.19, –0.18; *p* < 0.01), and BC (β = –1.61; 95% CI: –2.70, –0.51; *p* < 0.01).

**Table 3:** Estimated regression coefficients relating BAs to BOT-2 scores in models with and without adjustment for other variables [β (95% CI); *n* = 299].

Exposure measure	FMC	MC	BC	SA	TMC
Before adjustment										
As*a*		–1.94 (–3.55, –0.35)**		–0.70 (–2.07, 0.66)		–1.38 (–2.50, –0.26)**		0.07 (–0.63, 0.78)		–3.96 (–7.38, –0.55)*
Mn*a*		–2.93 (–7.02, 1.16)		–3.61 (–7.07, –0.16)*		–0.39 (–3.27, 2.47)		–1.37 (–3.16, 0.41)		–8.32 (–17.02, 0.39)^#^
Se*b*		1.55 (0.40, 2.70)**		1.57 (0.60, 2.55)**		0.15 (–0.66, 0.97)		0.26 (–0.25, 0.77)		3.54 (1.10, 6.00)**
After adjustment*c*										
As*a*		–1.68 (–3.19, –0.18)*		–0.49 (–1.73, 0.76)		–1.61 (–2.70, –0.51)**		0.15 (–0.57, 0.86)		–3.63 (–6.72, –0.54)*
Mn*a*		1.62 (–2.53, 5.77)		1.03 (–2.40, 4.47)		2.03 (–0.99, 5.06)		–0.66 (–2.64, 1.31)		4.02 (–4.52, 12.56)
Se*b*		0.79 (–0.41, 1.98)		1.02 (0.04, 1.99)*		0.30 (–0.58, 1.66)		0.09 (–0.48, 0.66)		2.17 (–0.30, 4.63)^†^
**a**Log transformed. **b**Square transformed. **c**The adjusted variables were sex, school attendance, head circumference, mother’s intelligence (WASI), plasma ferritin, BMn, BSe, and BPb. *R*^2^ values for adjusted models for FMC, MC, BC, SA, and TMC were 0.18, 0.22, 0.12, 0.03, and 0.24, respectively. *p < 0.05, **p < 0.01, ^#^*p* = 0.06.										

**Figure 1:**
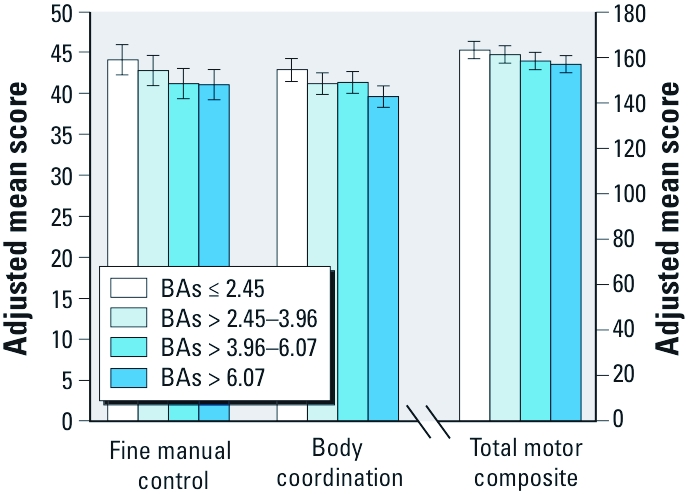
Covariate-adjusted mean scores by BAs quartiles for FMC, BC, and TMC scores. Models were adjusted for school attendance, head circumference, mother’s intelligence (WASI), plasma ferritin, BPb, BSe, and BMn. Significance levels: FMC (*p* = 0.07), BC (*p* < 0.05) and TMC (*p* = 0.1). Difference scales were used for adjusted mean scores for TMC.

Associations between As exposure and motor function were consistent with those observed for BAs when we defined exposure based on water, urine, or nail measures (Table 4). Figure 1 illustrates inverse dose–response associations between quartiles of BAs and adjusted mean TMC, FMC, and BC scores.

**Table 4:** Estimated regression coefficients relating measures of As exposure to BOT-2 scores [β (95% CI); *n* = 299)].

Exposure measure	FMC	MC	BC	SA	TMC
WAs (μ/L)		–0.54 (–1.03, –0.05)*		–0.15 (–0.52, 0.30)		–0.43 (–0.77, –0.06)*		–0.11 (–0.28, 0.18)		–1.18 (–2.13, –0.10)*
UAs (μ/L)		–1.03 (–2.45, 0.39)		–0.73 (–1.89, 0.44)		–1.43 (–2.67, –0.61)*		–0.19 (–0.86, 0.48)		–3.59 (–6.50, –0.68)**
UAs (g creatinine/L)		–0.88 (–2.28, 0.51)		–0.76 (–1.91, 0.38)		–1.60 (–2.61, –0.60)**		–0.16 (–0.83, 0.49)		–3.42 (–6.27, –0.57)*
NAs (μg/g)		–0.84 (–2.20, 0.50)		–0.68 (–1.80, 0.42)		–1.86 (–2.83, –0.89)**		–0.38 (–1.02, 0.25)		–3.77 (–6.52, –1.03)**
The models are adjusted for sex, school attendance, head circumference, mother’s intelligence (WASI), BPb, BSe, Bas, and BMn measures. Exposure values are log transformed. *p < 0.05, **p < 0.01.										

*Associations between BSe, BMn, and BPb and motor function.* BSe was significantly positively related to TMC (β = 3.54; 95% CI: 1.10, 6.00; *p* < 0.01), FMC (β = 1.55; 95% CI: 0.40, 2.27; *p* < 0.01), and MC (β = 1.57; 95% CI: 0.60, 2.55; *p* < 0.01) in unadjusted models. The positive association between BSe and TMC (β = 2.72; 95% CI: 0.11, 5.33; *p* < 0.05), FMC (β = 1.24; 95% CI: 0.01, 2.46; *p* < 0.05), and MC (β = 1.26; 95% CI: 0.22, 2.20; *p* < 0.01) remained significant when adjusted for BAs, BMn, and BPb. In addition, the positive association between BSe and MC remained significant (β = 1.02; 95% CI: 0.40, 1.99; *p* < 0.01) when adjusting for both other exposures and sociodemographic contributors. On the other hand, we found no significant associations between BMn or BPb and motor function (data not shown). We found no interaction with As exposure variables (all *p*-values > 0.10).

## Discussion

A primary strength of this study is the use of a well-standardized multidomain measure of children’s motor functioning (Cairney et al. 2009; Deitz et al. 2007; Rodger et al. 2003; Wang TN et al. 2009; Wilson et al. 1995). In this study, we observed an adverse association between As exposure and motor function in children. We found inverse associations between markers of As exposure and overall motor function regardless of the exposure marker employed (BAs, WAs, UAs, or NAs). In particular, As exposure was associated with decreases in FMC and BC. In contrast, increasing levels of BSe were positively associated with manual control (MC). In addition, maternal and child characteristics showed associations in the expected directions with motor function scores, attesting to the validity of the BOT-2 in our study population. However, we observed no association between Mn exposure and motor function among children in this study.

Among children exposed to As, studies from different parts of the world have documented poorer cognitive function, reflected in poorer memory skills, slower processing speed, deficits in verbal skill, and lower IQ (Calderon et al. 2001; von Ehrenstein et al. 2007; Wang et al. 2007; Wasserman et al. 2004, 2007). Currently, however, very little information is available on the mechanism(s) of As neurotoxicity in human.

*Adverse association between As and motor function.* Recent animal studies have reported a dose-dependent accumulation of As in many parts of the brain, including cerebral cortex, thalamus, cerebellum, pons, striatum, basal ganglia, and pituitary (Sanchez-Pena et al. 2010; Wang Y et al. 2009). The cortex, basal ganglia, and cerebellum all play important roles in cognition, memory, language development, and control and coordination of motor function. Both biochemical and morphologic changes in brain regions that are linked to cognitive and motor performance have been reported after dietary exposure to As in animal studies (Dhar et al. 2007; Haider and Najar 2008; Mishra and Flora 2008; Rao and Avani 2004; Samuel et al. 2005; Wang Y et al. 2009). For example, oxidative injury has been found in the cerebral cortex of rats (Haider and Najar 2008; Mishra and Flora 2008), as have deficits in cerebral nitric oxide production, important for neuronal transmission in the brain (Zarazua et al. 2006). Damage to Purkinje cells of the cerebellum has also been linked to As exposure (Dhar et al. 2007). Interestingly, an elevated level of As in cerebellar tissues has been associated with impaired performance on the Morris water maze test in mice (Wang Y et al. 2009). Taken together, these diverse findings suggest that As-induced changes in these brain regions may contribute to deficits in motor coordination.

*Association between Mn and motor function.* We did not detect significant associations between Mn exposure and motor function. Prior studies of Mn-related motor disturbances are based largely on occupational exposure in adults, involving lengthy periods of inhalation at very high levels (Cook et al. 1974; Roels et al. 1999). Past studies of children have linked Mn exposure to hyperactive (Bouchard et al. 2007) and classroom behavior (Khan et al. 2011), deficits in memory, poorer intellectual functioning (Bouchard et al. 2011; Menezes-Filho et al. 2011; Wasserman et al. 2006), and poorer learning (Collipp et al. 1983; Pihl and Parkes 1977).

Very little information has been available on the effects of Mn on children’s motor skills, although Takser et al. (2003) observed a negative relationship between cord blood Mn levels and visual-motor tasks from the McCarthy test (McCarthy 1972) that relied on hand movement (i.e., copying block designs) in a small (*n* = 63) sample of 3-year-old boys (Takser et al. 2003). Only scant data link Mn to motor function in animal studies. Deficits in spatial memory and decreased manual dexterity have been noted in monkeys injected with manganese sulfate (Schneider et al. 2006); delayed neurodevelopment has been reported in newborn rat pups orally supplemented with Mn (Tran et al. 2002). In both cases, extrapolations to human populations are problematic because doses studied may have little or no relevance to environmental exposure levels (Schneider et al. 2006; Tran et al. 2002).

*Protective association between Se and motor function.* Se is an essential nutrient, and inadequate Se intake is associated with adverse health outcomes—for example, Keshan’s disease (Lei et al. 2010). Increasing levels of BSe were associated with better motor function. Lower plasma Se levels have been associated with decreased motor coordination among patients with Parkinson’s disease (Richwine et al. 2005; Shahar et al. 2010). In mice reared in specific-pathogen-free conditions and fed a diet high in Se, improved psychomotor function has been observed (Richwine et al. 2005). In a rat model of parkinsonism, induced by exposure to 6-hydroxydopamine, pretreatment with sodium selenite showed evidence of a protective effect (Zafar et al. 2003). Collectively, these reports are consistent with a protective effect of Se on central nervous system functioning.

A protective role of Se in As-exposed populations has also been reported. Individuals in the HEALS cohort with higher BSe are less likely to develop As-induced skin lesions (Chen et al. 2007) and more likely to demonstrate adequate As metabolism (Pilsner et al. 2011). Earlier reports suggest antagonistic roles between As and Se (Levander 1977), such that As and Se may reduce each other’s toxicity by affecting biotransformation, distribution, and excretion (Csanaky and Gregus 2003; Kenyon et al. 1997). Moreover, among arsenicosis patients in China, dietary supplementation with Se was associated with a 73% decrease in hair levels of As (Wang et al. 2001). Consistent with this body of work, we observed a significant negative correlation between BSe and BAs.

The Se content of the Bangladeshi rural diet is lower than that in the United States. Dietary Se intake among rural Bangladeshi adults is estimated to be 17–20 μg/day based on U.S. Department of Agriculture tables for Se content in foods (Spallholz et al. 2004, 2005). In the HEALS cohort the average BSe of adults and children were 150 μg/L and 106 μg/L, respectively, comparable to those reported in developed countries (Combs 2001).

*Study limitations.* Study participants were healthy school children who were attending school regularly, so our findings cannot be generalized to all children, even in developing nations. Second, because no test of motor skill has been normalized in Bangladesh children, we made use of standardized motor scores that are based on U.S. norms. Village life in Bangladesh affords extensive and diverse opportunity for motor development (although activities are far less structured than in the United States), so use of U.S. norms is appropriate. The expectable associations with both socioeconomic and anthropometric markers (e.g., school attendance, head circumference) in this population provide support for our use of standardized scores.

*Public health importance.* Although children whose motor difficulties are severe enough to warrant a diagnosis of developmental coordination disorder (American Psychiatric Association 1994) obviously face challenges in home and school settings, three studies have found functional difficulties in children whose motor problems are less marked (Dewey et al. 2002; Rodger et al. 2003; Wang TN et al. 2009). We did not assess whether children with lower motor scores faced functional limitations in their daily activities at home or school, although the FMC subscale includes items such as cutting, mazes, and figure copying and was particularly affected by As exposure. These tasks may be assumed to affect aspects of functioning in the school setting.

## Conclusions

By design, half of the children in the study were consuming water with As lower than the World Health Organization recommended cutoff (< 10 μg/L). Our research demonstrates that exposure to As in drinking water at relatively low concentrations is related to children’s lower scores on a standardized test of motor skill. We also demonstrated a possible beneficial role of Se in this population. Our findings add a new sense of urgency to mitigate As exposures around the world.

## References

[r1] American Psychiatric Association (1994). Diagnostic and Statistical Manual of Mental Disorders (DSM). 4th ed.

[r2] Bouchard M, Laforest F, Vandelac L, Bellinger D, Mergler D. (2007). Hair manganese and hyperactive behaviors: pilot study of school-age children exposed through tap water.. Environ Health Perspect.

[r3] Bouchard MF, Sauve S, Barbeau B, Legrand M, Brodeur ME, Bouffard T (2011). Intellectual impairment in school-age children exposed to manganese from drinking water.. Environ Health Perspect.

[r4] Bruininks RH, Bruininks BH (2005).

[r5] Cairney J, Hay J, Veldhuizen S, Missiuna C, Faught BE (2009). Comparing probable case identification of developmental coordination disorder using the short form of the Bruininks-Oseretsky test of motor proficiency and the Movement ABC.. Child Care Health Dev.

[r6] Calderon J, Navarro ME, Jimenez-Capdeville ME, Santos-Diaz MA, Golden A, Rodriguez-Leyva I (2001). Exposure to arsenic and lead and neuropsychological development in Mexican children.. Environ Res.

[r7] Chen KL, Amarasiriwardena CJ, Christiani DC (1999). Determination of total arsenic concentrations in nails by inductively coupled plasma mass spectrometry.. Biol Trace Elem Res.

[r8] Chen Y, Hall M, Graziano JH, Slavkovich V, van Geen A, Parvez F (2007). A prospective study of blood selenium levels and the risk of arsenic-related premalignant skin lesions.. Cancer Epidemiol Biomarkers Prev.

[r9] Cheng ZY, Zheng Y, Mortlock R, van Geen A (2004). Rapid multi-element analysis of groundwater by high-resolution inductively coupled plasma mass spectrometry.. Anal Bioanal Chem.

[r10] Collipp PJ, Chen SY, Maitinsky S (1983). Manganese in infant formulas and learning disability.. Ann Nutr Metab.

[r11] Combs GF (2001). Selenium in global food systems.. Br J Nutr.

[r12] Cook D, Fahn S, Brait K. (1974). Chronic manganese intoxication.. Arch Neurol.

[r13] Csanaky I, Gregus Z. (2003). Effect of selenite on the disposition of arsenate and arsenite in rats.. Toxicology.

[r14] Das D, Chatterjee A, Mandal BK, Samanta G, Chakraborti D, Chanda B (1995). Arsenic in ground water in six districts of West Bengal, India: the biggest arsenic calamity in the world. Part 2. Arsenic concentration in drinking water, hair, nails, urine, skin-scale and liver tissue (biopsy) of the affected people.. Analyst.

[r15] Deitz JC, Kartin D, Kopp K (2007). Review of the Bruininks-Oseretsky test of motor proficiency, second edition (BOT-2).. Phys Occup Ther Pediatr.

[r16] Dewey D, Kaplan BJ, Crawford SG, Wilson BN (2002). Developmental coordination disorder: associated problems in attention, learning, and psychosocial adjustment.. Hum Mov Sci.

[r17] Dhar P, Mohari N, Mehra RD (2007). Preliminary morphological and morphometric study of rat cerebellum following sodium arsenite exposure during rapid brain growth (RBG) period.. Toxicology.

[r18] Hafeman DM, Ahsan H, Louis ED, Siddique AB, Slavkovich V, Cheng Z (2005). Association between arsenic exposure and a measure of subclinical sensory neuropathy in Bangladesh.. J Occup Environ Med.

[r19] Haider SS, Najar MS (2008). Arsenic induces oxidative stress, sphingolipidosis, depletes proteins and some antioxidants in various regions of rat brain.. Kathmandu Univ Med J.

[r20] Kenyon EM, Hughes MF, Levander OA (1997). Influence of dietary selenium on the disposition of arsenate in the female B6C3F1 mouse.. J Toxicol Environ Health.

[r21] Khan K, Factor-Litvak P, Wasserman GA, Liu X, Ahmed E, Parvez F (2011). Manganese exposure from drinking water and children’s classroom behavior in Bangladesh.. Environ Health Perspect.

[r22] Kim Y, Kim BN, Hong YC, Shin MS, Yoo HJ, Kim JW (2009). Co-exposure to environmental lead and manganese affects the intelligence of school-aged children.. Neurotoxicology.

[r23] Lei C, Niu X, Ma X, Wei J. (2010). Is selenium deficiency really the cause of Keshan disease?. Environ Geochem Health.

[r24] Levander OA (1977). Metabolic interrelationships between arsenic and selenium.. Environ Health Perspect.

[r25] McCarthy D (1972).

[r26] Menezes-Filho JA, Novaes Cde O, Moreira JC, Sarcinelli PN, Mergler D (2011). Elevated manganese and cognitive performance in school-aged children and their mothers.. Environ Res.

[r27] Miles LEM, Sipschitz DA, Bieter CP, Cook JD (1974). Measurement of serum ferritin by a 2-site immunoradiometric assay.. Anal Chem.

[r28] Mishra D, Flora SJ (2008). Differential oxidative stress and DNA damage in rat brain regions and blood following chronic arsenic exposure.. Toxicol Ind Health.

[r29] Mukherjee SC, Rahman MM, Chowdhury UK, Sengupta MK, Lodh D, Chanda CR, et al (2003).

[r30] Nixon DE, Mussmann GV, Eckdahl SJ, Moyer TP (1991). Total arsenic in urine: palladium-persulfate vs nickel as a matrix modifier for graphite furnace atomic absorption spectrophotometry.. Clin Chem.

[r31] Pihl RO, Parkes M (1977). Hair element content in learning disabled children.. Science.

[r32] Pilsner JR, Hall MN, Liu X, Ahsan H, Ilievski V, Slavkovich V (2011). Associations of plasma selenium with arsenic and genomic methylation of leukocyte DNA in Bangladesh.. Environ Health Perspect.

[r33] Pruszkowski E, Neubauer K, Thomas R. (1998). An overview of clinical applications by inductively coupled plasma mass spectrometry.. Atom Spectrosc.

[r34] Rahman MM, Chowdhury UK, Mukherjee SC, Mondal BK, Paul K, Lodh D (2001). Chronic arsenic toxicity in Bangladesh and West Bengal, India—a review and commentary.. J Toxicol Clin Toxicol.

[r35] Rao MV, Avani G (2004). Arsenic induced free radical toxicity in brain of mice.. Indian J Exp Biol.

[r36] Richwine AF, Godbout JP, Berg BM, Chen J, Escobar J, Millard DK (2005). Improved psychomotor performance in aged mice fed diet high in antioxidants is associated with reduced *ex vivo* brain interleukin-6 production.. Brain Behav Immun.

[r37] Rodger S, Ziviani J, Watter P, Ozanne A, Woodyatt G, Springfield E. (2003). Motor and functional skills of children with developmental coordination disorder: a pilot investigation of measurement issues.. Hum Mov Sci.

[r38] Roels HA, Ortega Eslava MI, Ceulemans E, Robert A, Lison D (1999). Prospective study on the reversibility of neurobehavioral effects in workers exposed to manganese dioxide.. Neurotoxicology.

[r39] Rosado JL, Ronquillo D, Kordas K, Rojas O, Alatorre J, Lopez P (2007). Arsenic exposure and cognitive performance in Mexican schoolchildren.. Environ Health Perspect.

[r40] Samuel S, Kathirvel R, Jayavelu T, Chinnakkannu P. (2005). Protein oxidative damage in arsenic induced rat brain: influence of DL-alpha-lipoic acid.. Toxicol Lett.

[r41] Sanchez-Pena LC, Petrosyan P, Morales M, Gonzalez NB, Gutierrez-Ospina G, Del Razo LM (2010). Arsenic species, AS3MT amount, and AS3MT gene expression in different brain regions of mouse exposed to arsenite.. Environ Res.

[r42] Schneider JS, Decamp E, Koser AJ, Fritz S, Gonczi H, Syversen T (2006). Effects of chronic manganese exposure on cognitive and motor functioning in non-human primates.. Brain Res.

[r43] Shahar A, Patel KV, Semba RD, Bandinelli S, Shahar DR, Ferrucci L (2010). Plasma selenium is positively related to performance in neurological tasks assessing coordination and motor speed.. Mov Disord.

[r44] Spallholz JE, Boylan LM, Palace V, Chen J, Smith L, Rahman MM (2005). Arsenic and selenium in human hair: a comparison of five countries with and without arsenicosis.. Biol Trace Elem Res.

[r45] Spallholz JE, Mallory Boylan L, Rhaman MM (2004). Environmental hypothesis: is poor dietary selenium intake an underlying factor for arsenicosis and cancer in Bangladesh and West Bengal, India?. Sci Total Environ.

[r46] Stroh A. (1988). Determination of Pb and Cd in whole blood using isotope dilution ICP-MS.. Atom Spectrosc.

[r47] Takser L, Mergler D, Hellier G, Sahuquillo J, Huel G. (2003). Manganese, monoamine metabolite levels at birth, and child psychomotor development.. Neurotoxicology.

[r48] Tran TT, Chowanadisai W, Crinella FM, Chicz-DeMet A, Lonnerdal B (2002). Effect of high dietary manganese intake of neonatal rats on tissue mineral accumulation, striatal dopamine levels, and neurodevelopmental status.. Neurotoxicology.

[r49] Tseng HP, Wang YH, Wu MM, The HW, Chiou HY, Chen CJ (2006). Association between chronic exposure to arsenic and slow nerve conduction velocity among adolescents in Taiwan.. J Health Popul Nutr.

[r50] van Geen A, Cheng Z, Jia Q, Seddique AA, Rahman MW, Rahman MM, et al (2007).

[r51] van Geen A, Cheng Z, Seddique AA, Hoque MA, Gelman A, Graziano JH (2005). Reliability of a commercial kit to test groundwater for arsenic in Bangladesh.. Environ Sci Technol.

[r52] von Ehrenstein OS, Poddar S, Yuan Y, Mazumder DG, Eskenazi B, Basu A (2007). Children’s intellectual function in relation to arsenic exposure.. Epidemiology.

[r53] Wang SX, Wang ZH, Cheng XT, Li J, Sang ZP, Zhang XD (2007). Arsenic and fluoride exposure in drinking water: children’s IQ and growth in Shanyin county, Shanxi province, China.. Environ Health Perspect.

[r54] Wang TN, Tseng MH, Wilson BN, Hu FC (2009). Functional performance of children with developmental coordination disorder at home and at school.. Dev Med Child Neurol.

[r55] Wang W, Yang L, Shaofan H, Jian’an T, Hairong L. (2001). Prevention of endemic arsenism with selenium.. Curr Sci.

[r56] Wang Y, Li S, Piao F, Hong Y, Liu P, Zhao Y. (2009). Arsenic down-regulates the expression of Camk4, an important gene related to cerebellar LTD in mice.. Neurotoxicol Teratol.

[r57] Wasserman GA, Liu X, Parvez F, Ahsan H, Factor-Litvak P, Kline J (2007). Water arsenic exposure and intellectual function in 6-year-old children in Araihazar, Bangladesh.. Environ Health Perspect.

[r58] Wasserman GA, Liu X, Parvez F, Ahsan H, Factor-Litvak P, van Geen A (2004). Water arsenic exposure and children’s intellectual function in Araihazar, Bangladesh.. Environ Health Perspect.

[r59] Wasserman GA, Liu X, Parvez F, Ahsan H, Levy D, Factor-Litvak P (2006). Water manganese exposure and children’s intellectual function in Araihazar, Bangladesh.. Environ Health Perspect.

[r60] Wasserman GA, Liu X, Parvez F, Factor-Litvak P, Ahsan H, Levy D (2011). Arsenic and manganese exposure and children’s intellectual function.. Neurotoxicology.

[r61] Wechsler D (1999).

[r62] Wilson BN, Polatajko HJ, Kaplan BJ, Faris P (1995). Use of the Bruininks-Oseretsky test of motor proficiency in occupational therapy.. Am J Occup Ther.

[r63] Wright RO, Amarasiriwardena C, Woolf AD, Jim R, Bellinger DC (2006). Neuropsychological correlates of hair arsenic, manganese, and cadmium levels in school-age children residing near a hazardous waste site.. Neurotoxicology.

[r64] Zafar KS, Siddiqui A, Sayeed I, Ahmad M, Salim S, Islam F (2003). Dose-dependent protective effect of selenium in rat model of Parkinson’s disease: neurobehavioral and neurochemical evidences.. J Neurochem.

[r65] Zarazua S, Perez-Severiano F, Delgado JM, Martinez LM, Ortiz-Perez D, Jimenez-Capdeville ME (2006). Decreased nitric oxide production in the rat brain after chronic arsenic exposure.. Neurochem Res.

